# Novel Strategies for Peptide-Based Vaccines in Hematological Malignancies

**DOI:** 10.3389/fimmu.2018.02264

**Published:** 2018-10-01

**Authors:** Uffe Klausen, Staffan Holmberg, Morten Orebo Holmström, Nicolai Grønne Dahlager Jørgensen, Jacob Handlos Grauslund, Inge Marie Svane, Mads Hald Andersen

**Affiliations:** ^1^Center for Cancer Immune Therapy, Herlev Hospital, Department of Hematology and Oncology, Herlev, Denmark; ^2^Department of Hematology, Herlev Hospital, Herlev, Denmark; ^3^Division of Immunology - T cells & Cancer, DTU Nanotech, Technical University of Denmark, Lyngby, Denmark; ^4^Department of Hematology, Zealand University Hospital, Roskilde, Denmark; ^5^Institute for Clinical Medicine, University of Copenhagen, Copenhagen, Denmark; ^6^Institute for Immunology and Microbiology, University of Copenhagen, Copenhagen, Denmark

**Keywords:** peptide vaccination, follicular lymphoma, multiple myeloma, myeloproliferative neoplasms, myelodysplastic syndrome, PD-1, cancer testis antigen, neo-antigens

## Abstract

Peptides vaccination is an interesting approach to activate T-cells toward desired antigens in hematological malignancies. In addition to classical tumor associated antigens, such as cancer testis antigens, new potential targets for peptide vaccination comprise neo-antigens including JAK2 and CALR mutations, and antigens from immune regulatory proteins in the tumor microenvironment such as programmed death 1 ligands (PD-L1 and PD-L2). Immunosuppressive defenses of tumors are an important challenge to overcome and the T cell suppressive ligands PD-L1 and PD-L2 are often present in tumor microenvironments. Thus, PD-L1 and PD-L2 are interesting targets for peptide vaccines in diseases where the tumor microenvironment is known to play an essential role such as multiple myeloma and follicular lymphoma. In myelodysplastic syndromes the drug azacitidine re-exposes tumor associated antigens, why vaccination with related peptides would be an interesting addition. In myeloproliferative neoplasms the JAK2 and CALR mutations has proven to be immunogenic neo-antigens and thus possible targets for peptide vaccination. In this mini review we summarize the basis for these novel approaches, which has led to the initiation of clinical trials with various peptide vaccines in myelodysplastic syndromes, myeloproliferative neoplasms, multiple myeloma, and follicular lymphoma.

## Introduction

**Figure 1:**
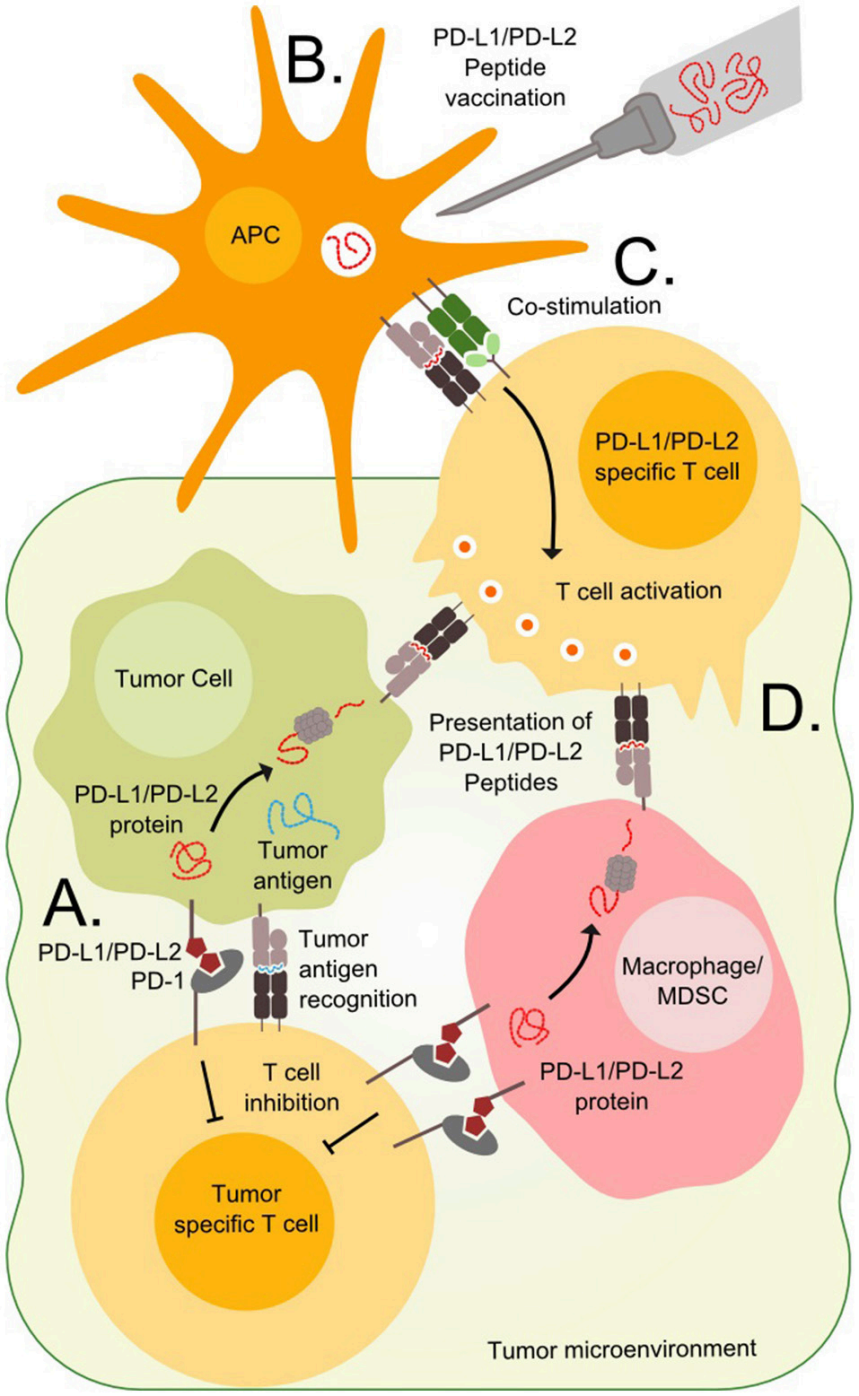
Targeting PD-L1 and PD-L2 expressing cells. **(A)** T cells in the tumor microenvironment often express PD-1 and are vulnerable to stimulation from the ligands PD-L1 or PD-L2 expressed on tumor cells or tumor infiltrating cells such as macrophages or Myeloid-derived suppressor cells (MDSC). **(B)** Immunogenic peptides derived from the PD-L1 and PD-L2 can be injected in the patients where they are endocytosed and processed by antigen presenting cells (APC). **(C)** The APCs present the peptides to T cells in the draining lymph node along with co-stimulatory signals, which are necessary for priming and optimal cytotoxicity. **(D)** Tumor cells, macrophages and MDSCs expressing PD-L1 and PD-L2 also present epitopes derived from these proteins on surface MHC molecules and are vulnerable to primed PD-L1 and PD-L2 specific T cells.

Cancer vaccine therapy is based on the principle of activating an immune response toward cancer cells. The concept dates back to the Nineteenth century when William Coley attempted to raise an immune response against cancer by exposing patients to bacterial extracts (1). In the view of modern research standards Coley's results are questionable, but since then the field has evolved immensely and modern therapeutic cancer vaccines induce potent anti-tumor immune responses. The field of therapeutic cancer vaccines involves a variety of methods including cellular vaccines, RNA/DNA based vaccines, viral vaccines, and peptide/protein vaccines described in detail by Gou et al. (2) Peptide vaccines hold the advantage of short production times and easy administration and will be the focus of this review. This method is based on peptides from selected tumor proteins that are injected into patients along with an immune activating adjuvant. After injection, the peptides are processed by antigen presenting cells and presented to T cells in the draining lymph node, as illustrated in Figures 1B,C. T cells recognizing the presented epitopes are primed to recognize cells expressing the target proteins, as these are presenting the epitopes on the cell surface. The vaccine field is fueled by the continuous discovery of targetable epitopes. Such epitopes are either neo-antigens, which are formed by somatic mutations that generate a novel mutant antigen, or non-mutated antigens that are overexpressed by the neoplastic cells. Unfortunately, therapeutic cancer vaccination has yet to show significant clinical impact. Limitations to this approach involves a variety of immune escape mechanisms including defected antigen presentation identified in many tumors and T cells unable to find or penetrate the tumors, which might be a minor issue in hematological malignancies as these by nature are less immune restricted than solid tumors (3). Another major limitation is the immunosuppressive mechanisms employed by tumor cells and regulatory cells in the tumor microenvironment (Figure 1A) (2). Immune checkpoints such as the PD-1/PD-L1 pathway inhibit activated T cells and thereby prevent an effective antitumor response. Monoclonal antibodies blocking these pathways known as checkpoint inhibitors allow the activated T cells to function regardless of the suppressive signals from the surroundings. Checkpoint inhibitors have proven effective in both solid and hematological cancers (4). However, not all tumors respond to checkpoint inhibitors and they are associated with serious side effects. Targeting the checkpoints through therapeutic vaccination offers a novel way to directly target regulatory pathways in the tumor microenvironment and potentially modify tolerance to tumor antigens. Like the checkpoint inhibitors the vaccine approach might relieve the immune suppression and potentiate anti-tumor T cell responses, but in addition, the vaccine may recruit activated T cells to the tumor site and promote epitope spreading when the target cells are killed. Addressing the immune regulatory mechanisms is essential to improve the outcomes of peptide vaccination.

In this mini review we summarize novel strategies to overcome immune suppression and enhance tumor recognition, which have led to clinical trials in myelodysplastic syndrome, myeloproliferative neoplasms, multiple myeloma, and follicular lymphoma.

## Targeting immune checkpoints in multiple myeloma

Multiple myeloma (MM) is a neoplastic disease of plasma cells with hallmarks including hypercalcemia, renal insufficiency, anemia, and bone lesions. In the recent years several new treatment options have become available, which has improved the median survival. However, the disease is still incurable. All cases of MM are preceded by the precursor state monoclonal gammopathy of undetermined significance (MGUS) and some patients progress via an intermediate state termed smoldering multiple myeloma (SMM) (5). Since the majority of genetic mutations are already present in the precursor states, changes in the microenvironment are believed to impact the risk of progression (6). The microenvironment in MM is severely immunosuppressive (7), and decreased humoral and cellular immune responses to viral and neoplastic epitopes in patients with MGUS and SMM are risk factors for progression to MM (8). Progression from MGUS to MM is also correlated to the expression level of the immune checkpoint molecule programmed death ligand 1 (PD-L1) on MM cells (8). PD-L1 interacts with the molecule PD-1 on T cells and serves as a powerful negative regulatory signal, which plays a major role in the normal physiologic maintenance of immune self-tolerance, reviewed in Keir et al (9). In symptomatic MM, T cells and natural killer (NK) cells in the tumor microenvironment display increased amounts of PD-1, and MM-cells, osteoclasts and dendritic cells demonstrate elevated levels of PD-L1 (10–16). One study showed that PD-L1 is variably expressed on clonal plasma cells in newly diagnosed MM patients (17). The PD-1/PD-L1 pathway not only promotes the progression of myeloma indirectly by immune evasion; bone marrow stromal cells induce myeloma cells to express PD-L1, which results in increased tumor cell proliferation and reduced susceptibility to anti-myeloma chemotherapy (18). Extramedullary plasmacytomas from patients with late stage MM are characterized by increased expression of PD-L1 (19). Furthermore, the level of PD-1 on T cells is inversely correlated with overall survival (20). Additionally, patients display increased levels of PD-L1 on myeloma cells at relapse or when refractory to treatment, and is associated with an aggressive disease phenotype (21). Increased numbers of T cells with upregulated PD-1 and an exhausted immune phenotype is identified in patients that relapse after high-dose chemotherapy followed by allogeneic hematopoietic stem cell transplantation (HDT-ASCT), indicating that the PD-1/PD-L1 axis could be an important determinant of early relapse after HDT-ASCT (22).

We have characterized T cells in cancer patients that are able to recognize peptides derived from PD-L1 protein, and demonstrated that specific T cells isolated and expanded from these patients are able to recognize and kill PD-L1 expressing cells (23, 24). PD-L1 specific T cells target both tumor cells as well as PD-L1 expressing cells in the microenvironment (Figure 1D) (25, 26). Furthermore, stimulation of T cell cultures with PD-L1 peptide was *in vitro* shown to boost the antineoplastic effect of a dendritic cell (DC)-vaccine (27). This effect is likely based on the ability of PD-L1 specific T cells to kill regulatory PD-L1 positive cells in the cell culture, consequently leading to an attenuated immune regulation.

Based on these observations, we have initiated a phase I study testing safety and efficacy of PD-L1 peptide vaccination as a monotherapy consolidation after HDT-ASCT in patients with MM. Furthermore, we are initiating a vaccination study with PD-L1 peptide for patients with SMM. Of note, monotherapy with the anti PD-1 monoclonal antibody (mAb) nivolumab did not show effect in MM (28). Several combination studies of PD-1 specific mAbs have been halted by the Food and Drug Administration (FDA) due to increased mortality in the experimental arms. The halt has recently been lifted on several studies, but the difficulties using anti-PD-1 mAbs for MM underline the need for development of alternative approaches to target the PD-1/PD-L1 pathway in MM.

## Targeting immune checkpoints in follicular lymphoma

Follicular lymphoma (FL) is an incurable disease characterized by waxing and waning courses of the disease and is often monitored without the need for active treatment. Over time the disease expands and there is a substantial risk of transformation to more aggressive lymphomas. The mainstay treatment is chemotherapy and anti-CD20 mAbs. Since FL is an indolent disease, it is believed to be ideal for vaccination therapy, which has been explored in FL, in the form of anti-idiotype cancer vaccines. So far this approach has failed to show clinical benefit when tested against placebo or chemotherapy in phase III trials (29–31). There are many possible reasons for the lack of success in these trials, but the immunosuppressive microenvironment in FL is a probable explanation. A gene expression study in FL revealed that the gene signature from regulatory immune cells was an independent adverse prognostic factor (32). Another study looked at the gene expression of specific immunosuppressive proteins in the microenvironment and found 24 out of 54 to be upregulated in FL compared to healthy tissue (33). PD-L1 and programmed death ligand 2 (PD-L2) were among the upregulated genes, which also was confirmed by immunohistochemistry. Both PD-L1 and PD-L2 play a role in immune suppression and contribute to the reduced cytotoxic potential of effector T cells (34). In FL PD-L1 expression has also been identified on tumor-infiltrating macrophages (35).

The clinical relevance of the PD-1 pathway was investigated in a phase I checkpoint inhibition trial, where heavily treated FL patients were treated with the PD-1 blocking mAb Nivolumab as monotherapy. 4 out of 10 had an objective response and one achieved complete response (CR) (28), indicating that the PD-1/Ligand pathway could be important for successful vaccination therapy. As mentioned above, cytotoxic PD-L1 specific T cells can be expanded in cultures by stimulation with PD-L1 derived peptides. Likewise, immunogenic PD-L2 epitopes have been identified, and spontaneous immune responses against these epitopes have been observed in cancer patients (36). Additionally, PD-L2 specific T cells are cytotoxic to PD-L2 expressing tumor cells. Based on these findings and additional unpublished data, we are conducting a phase I vaccination trial with PD-L1 and PD-L2 derived peptides in relapsed FL as maintenance after chemotherapy (NCT03381768). This vaccine is primarily targeting the PD-L1 and PD-L2 positive tumor infiltrating macrophages known to stimulate tumor vascularization and moreover have been correlated with disease transformation and poor prognosis (37, 38). Furthermore, the macrophages seem to have a lymphoma propagating role by secretion of IL15 (39). Thus, by targeting PD-L1 and PD-L2 expressing tumor- and regulatory cells in FL, we hope to shift the immunological balance toward tumor elimination.

## Targeting cancer testis antigens in myelodysplastic syndrome

**Figure 2:**
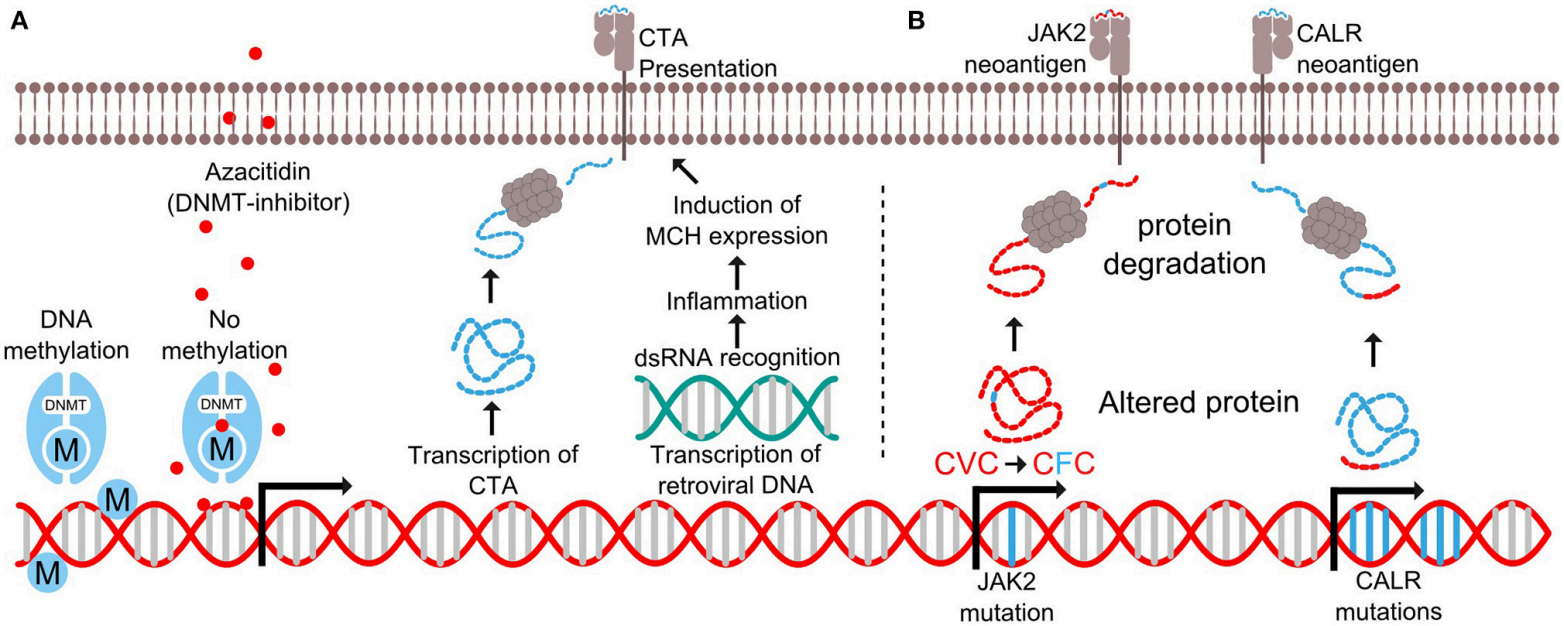
Expression of antigens in myelodysplastic syndrome and myeloproliferative neoplasms. **(A)** DNA methyltransferase (DNMT) add methyl (M) groups to parts of the genome to prevent transcription. The drug azacitidine binds to cytidine nucleotides where it covalently attaches to DNMT to prevent further methylation. This results in the transcription of otherwise suppressed genes such as cancer testis antigens (CTA) and retroviral DNA. The CTA are processed as proteins and presented by MHC molecules on the cell surface, while the double stranded RNA (dsRNA) trigger intracellular pattern recognition receptors causing inflammation and increased MHC expression. **(B)** Mutations in the *JAK2* gene results in the substitution of valine (V) to phenylalanine (F) in position 617 of the JAK2 protein. This results in the generation of a mutant antigen. Likewise, the *CALR* exon 9 mutations generate a novel mutant C-terminus in the CALR protein, thus generating several mutant antigens.

Myelodysplastic syndrome (MDS) is a malignant disorder characterized by clonal expansion of mutated myeloid precursor cells, resulting in an accumulation of blasts in the bone marrow and cytopenia due to ineffective hematopoiesis. MDS responds poorly to chemotherapy, and the only curative treatment is allogeneic HSCT (allo-HSCT), which most often is not feasible due to the high treatment related mortality. Hypomethylating agents (HMA), such as azacitidine or decitabine, are standard therapies for patients with high-risk MDS, who are not eligible for an allo-HSCT. HMAs works by incorporating themselves into the DNA by competitively binding at cytidine nucleotides. After DNA incorporation, the drug covalently attaches to DNA methyltransferase (DNMT), resulting in a loss of methylation and subsequently re-expression of the affected genes as the cell divides (Figure 2A) (40).

Several possible synergies may be achieved by combining HMA with therapeutic cancer vaccination. Firstly, a group of genes called cancer testis antigens (CTA) not usually expressed in healthy tissue due to gene methylation, has been found to be expressed by neoplastic cells (41). Treatment with HMA has shown to enhance the expression of CTA (42–46), while not affecting the expression in healthy tissue (47–49). Since healthy cells do not express CTA, the immune system has not developed central tolerance to these antigens, and they can be exploited as targets for immunotherapy. Secondly, HMA induces transcription of DNA from endogenous retroviruses resulting in an inflammatory response in tumor cells (50–53). Double stranded RNA from the viruses activates viral defense pathways, which causes the cell to produce interferon's and upregulate HLA class I molecules (Figure 2A). This inflammatory response makes the cancer cells more susceptible to immune mediated killing. Thirdly, the bone marrow of MDS patients has an immunosuppressive microenvironment with an increased amount of myeloid derived suppressor cells (MDSCs) (54). HMA has been shown to deplete MDSCs (55), thus potentially making it easier for T cells to exert an effective tumor-specific immune response.

Vaccination against CTA as monotherapy has previously been tested in many cancer types with varying success (56–58), and trials combining CTA-derived epitopes with HMA are now emerging (59, 60). In NCT02750995 we are targeting four CTAs (NY-ESO-1, PRAME, MAGE-A3, and WT-1) in combination with azacitidine, and another study is investigating a dendritic cell directed vaccine targeting NY-ESO-1 in combination with decitabine and a PD-1 checkpoint inhibitor (NCT03358719). The use of checkpoint inhibitors is expected to further enhance the potency of the combination therapy, since HMA also induces upregulation of PD-L1 on tumor cells and PD-1 on T cells (61, 87).

## Targeting neo-antigens in myeloproliferative neoplasms

Chronic myeloproliferative neoplasms (MPN) are cancer diseases of the hematopoietic stem cells of the bone marrow and are characterized by an increased production of peripheral blood cells. MPNs display a very homogenic mutational landscape, as 50% of patients harbor the Janus Kinase 2 (*JAK2*)V617F driver mutation (62, 63), and 20–25% have a driver mutation in exon 9 of the calreticulin (*CALR*) gene (64, 65). Recently, both of these mutations were shown to be targets of specific T cells (Figure 2B) (66–68). These findings have opened an avenue for therapeutic cancer vaccination with peptides derived from the *JAK2*- or *CALR*-mutations for patients with MPN. However, MPN-patients display several immune-regulatory mechanisms that may attenuate the tumor specific immune response induced by vaccination. Wang et al. showed that patients with MPN have increased numbers of MDSC in peripheral blood, and that mononuclear cells from MPN-patients express increased amounts of the immunoregulatory enzyme arginase-1 compared to healthy donors (69). Additionally, MDSCs from MPN patients are more suppressive to T cells compared to MDSCs from healthy donors. Prestipino and colleagues recently showed that the *JAK2*V617F-mutation enhances PD-L1 expression in mutant cells through activation of STAT3 and STAT5 (70). As described above, both arginase-I and PD-L1 are targets of specific T cells (23, 24, 71), and the immune mediated killing of arginase-I and PD-L1 expressing cells is believed to enhance the tumor specific immune response (72). Recently, strong and frequent spontaneous T-cell responses against both PD-L1 and arginase-1 were detected in patients with MPN (73, 74). We hypothesize that enhancing these already existing anti-regulatory T-cell responses through therapeutic cancer vaccination with arginase-I and PD-L1 derived epitopes can boost the neo-antigen specific immune response induced by vaccination with JAK2/CALR-mutant epitopes. This method of combinatorial cancer vaccination targeting both driver mutations and immunoregulation could potentially break the immune evasion leading to anti-tumor immunity and clinical effect. Another means to enhance the anti-tumor immune response would be to combine JAK2/CALR-vaccines with PD-1 specific mAbs, as treatment with these drugs have been shown to enhance the amount of neo-antigen specific T cells in peripheral blood (75).

Apart from the obvious combination of JAK2/CALR mutant vaccines with immune checkpoint blocking antibodies, the combination of vaccines with interferon-alpha (IFN-α) is a most interesting option. IFN-α is a potent immunostimulatory cytokine and has been used for years for the treatment of MPN (76). IFN-α has been shown to induce complete hematological responses and major molecular remissions in a substantial proportion of patients (77–79). Concurrently, treatment with IFN-α induces marked alterations in immune cell subsets and in the expression of HLA-related genes (80–83), and the mechanism beyond the clinical effect of IFN-α is believed to rely partially on the induction of an anti-tumor immune response (84). Previous reports on therapeutic cancer vaccination in other malignancies have underscored the importance of a low tumor burden at the time of vaccine initiation in order to obtain a proper clinical response (85). As IFN-α is the only drug, which is able to reduce the tumor burden in a substantial part of the patients, it is most apparent to reduce the tumor burden with IFN-α, and after attainment of a major molecular remission, initiate therapeutic cancer vaccination against the targets described above. This could hopefully eradicate the malignant clone and ultimately cure the patient. However, as exposure of cells to interferon increases the expression of PD-L1 on the exposed cells it could be worthwhile to explore the combination of neo-antigen vaccines and IFN-α with either PD-1 blocking mAbs and/or PD-L1 vaccine in order to counteract the increased amounts of PD-1 ligands induced by IFN-α treatment (86).

## Conclusion

The trials described above represent novel approaches to overcome some of the challenges in peptide vaccination including the suppressive mechanisms protecting the tumor cells from an effective anti-tumor immune response. Targeting the immune checkpoints such as the PD-1 ligands or other immune suppressive molecules such as arginase-1 could shift the immunological balance in the tumor microenvironment and ultimately induce an adequate anti-tumor immune response—a strategy that is currently being explored in FL and MM. Combining this approach with tumor specific antigens such as the neoantigens described in MPN could further enhance the anti-tumor response. Finally, combining vaccination against shared antigens, such as CTA, with HMA treatment in MDS is a promising approach to increase immunogenicity of the malignant cells. If the peptide vaccines prove safe and ultimately effective, they will become welcome additions to the toxic treatment options currently available for patients with hematological cancers.

## Ethics statement

All undergoing studies mentioned in the review are approved by the ethical committee of the capital region of Denmark and conducted according to national ethical guidelines and the Helsinki declaration.

## Author contributions

MA, IS, and UK contributed to the conception and design of the review. UK wrote the first draft of the manuscript and provided the figures. SH, MH, NJ, and JG wrote sections of the manuscript. All authors contributed to manuscript revision, read and approved the submitted version.

### Conflict of interest statement

MA and MH have filed a patent application on the *JAK2* and *CALR* mutations for therapeutic cancer vaccination. MA has filed patent applications on the use of PD-L1, PD-L2, and arginase-1 for therapeutic cancer vaccines. The rights of the patents have been transferred to the Capital Region and Zealand Region according to Danish law on inventions made at public research institutions. The capital region has licensed some of these patents to the company IO Biotech ApS. MA is a shareholder and board member of the IO Biotech ApS, which has the purpose of developing immune-modulating vaccines for cancer treatment. The remaining authors declare that the research was conducted in the absence of any commercial or financial relationships that could be construed as a potential conflict of interest.
